# Partial and Transient Clinical Response to Omalizumab in IL-21-Induced Low STAT3-Phosphorylation on Hyper-IgE Syndrome

**DOI:** 10.1155/2019/6357256

**Published:** 2019-07-04

**Authors:** Cesar Daniel Alonso-Bello, María del Carmen Jiménez-Martínez, María Eugenia Vargas-Camaño, Sagrario Hierro-Orozco, Mario Alberto Ynga-Durand, Laura Berrón-Ruiz, Julio César Alcántara-Montiel, Leopoldo Santos-Argumedo, Diana Andrea Herrera-Sánchez, Fernando Lozano-Patiño, María Isabel Castrejón-Vázquez

**Affiliations:** ^1^Immunology and Allergy Department, Centro Médico Nacional 20 de Noviembre, Instituto de Seguridad y Servicios Sociales de los Trabajadores del Estado, Mexico; ^2^Faculty of Medicine, Universidad Nacional Autónoma de México, Mexico; ^3^Dermatology Department, Centro Médico Nacional 20 de Noviembre, Instituto de Seguridad y Servicios Sociales de los Trabajadores del Estado, Mexico; ^4^Universidad Nacional Autónoma de México, Facultad de Estudios Superiores Zaragoza, Facultad de Medicina, Mexico; ^5^Laboratorio de Inmunidad de Mucosas, Sección de Investigación y Posgrado, Escuela Superior de Medicina, Instituto Politécnico Nacional, Mexico City, Mexico; ^6^Immunodeficiencies Research Unit, Instituto Nacional de Pediatria, Secretaría de Salud, Mexico; ^7^Department of Molecular Biomedicine, CINVESTAV-IPN, Mexico

## Abstract

Hyper-IgE syndrome (HIES) is a rare primary immunodeficiency characterized by elevated levels of immunoglobulin E (IgE), eczematous dermatitis, cold abscesses, and recurrent infections of the lung and skin caused by* Staphylococcus aureus*. The dominant form is characterized by nonimmunologic features including skeletal, connective tissue, and pulmonary abnormalities in addition to recurrent infections and eczema. Omalizumab is a humanized recombinant monoclonal antibody against IgE. Several studies reported clinical improvement with omalizumab in patients with severe atopic eczema with high serum IgE level. We present the case of a 37-year-old male with HIES and cutaneous manifestations, treated with humanized recombinant monoclonal antibodies efalizumab and omalizumab. After therapy for 4 years, we observed diminished eczema and serum IgE levels.

## 1. Introduction

Hyper-IgE syndrome (HIES) is a rare primary immunodeficiency characterized by elevated levels of immunoglobulin E (IgE), eczematous dermatitis, cold abscesses, and recurrent infections of the lung and skin caused by* Staphylococcus aureus*. The diagnosis of hyper-IgE syndrome is syndromic and the most frequent laboratory meetings are eosinophilia (greater than 1500/ml) and elevated levels of IgE, which typically are above 2000 IU/ml. Leukocytes in blood and serum immunoglobulin levels IgM, IgG, and IgA are frequently normal, but they have been found in some cases of deficiencies immunoglobulins. There are two main forms of HIES: a form characterized by mutations in signal transducer and activator of transcription 3 gene (*STAT3*) with autosomal dominant inheritance (AD) (the Online Mendelian Inheritance in Man® (OMIM®) number registered in database is #147060) and a mutation in Dedicator of cytokinesis 8 (*DOCK8*) with autosomal recessive inheritance (AR), OMIM #243700, both in humans and mice. The dominant form is characterized by nonimmunologic features including skeletal abnormalities (scoliosis, retained primary teeth, and fractures with minor trauma), connective tissue, and pulmonary abnormalities in addition to recurrent infections and eczema. In contrast, the recessive form lacks skeletal abnormalities and has marked viral infections caused by molluscum contagiosum and herpes simplex virus (HSV) and neurologic complications [1]. The DOCK8 immunodeficiency syndrome (DIDS) is the predominant form of HIES autosomal recessive (AR-HIES). DOCK8 which is highly expressed in the immune system is a member of a poorly characterized family of atypical guanine dinucleotide exchange factors for Rho family GTPases. The 50-80% of these patients develop severe allergies such as asthma, anaphylaxis, and food allergy. In particular, CD8+ T cells from DIDS patients not only are decreased in number but also fail to expand normally in vitro after stimulation and produce less antiviral cytokines, which increases susceptibility to viral infections like herpes simplex virus (HSV), human papillomavirus (HPV), molluscum contagious (MCV), and varicella-zoster virus (VZV). Some patients have impaired humoral immunity, decreasing the number of immunoglobulins. DIDS patients are also highly prone to malignancy, with 10% to 36% of patients developing cancers in late childhood to early adulthood like squamous cell carcinomas, and lymphomas, including Burkitt lymphoma or Epstein Barr Virus (EBV) diffuse large B cell lymphoma, predominate [2, 3]. Since the discovery in 2009 that loss-of-function mutations in* DOCK8 *underlie AR-HIES, an estimated >100 patients worldwide have been identified. FOXP3 regulatory T cells that produce IL-10 play an essential role in regulating allergic inflammation. There are two types of FOXP3 regulatory T cells: the natural and induced.* FOXP3* gene mutations produce immune deregulation with enteropathy, autoimmune diabetes, thyroiditis, food allergies, atopic dermatitis, and elevated levels of IgE. Cytokines produced by dendritic cells (DC) play a role in the induction and maintenance of T cell tolerance. IL-10 signaling-DC is needed for the generation of tolerogenic DC and induced regulatory T cells (iTreg) which maintain an appropriate balance Th1/Th2. In patients with HIES, signal transduction mediated by IL10-DCs is impaired, which predisposes to allergic manifestations. This generation of induced FOXP3 regulatory T cells is impaired in patients with HIES [4]. We describe a case of partial clinical response to omalizumab in IL-21-induced low STAT3-phosphorylation on a patient with HIES and very high levels of total serum IgE.

## 2. Case Presentation

**Figure 1:**
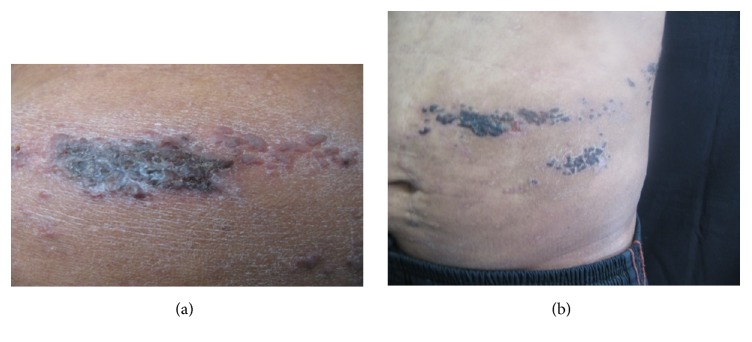
Cutaneous lesions located in left costal dermatome, characterized by multiple necrotic lesions.

**Figure 2:**
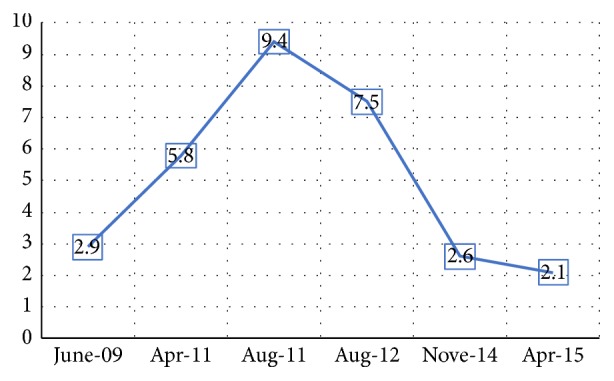
Comparison between CRP levels during application of omalizumab. The variation of protein levels does not correlate with the clinical manifestations of the patient and IgE levels.

**Figure 3:**
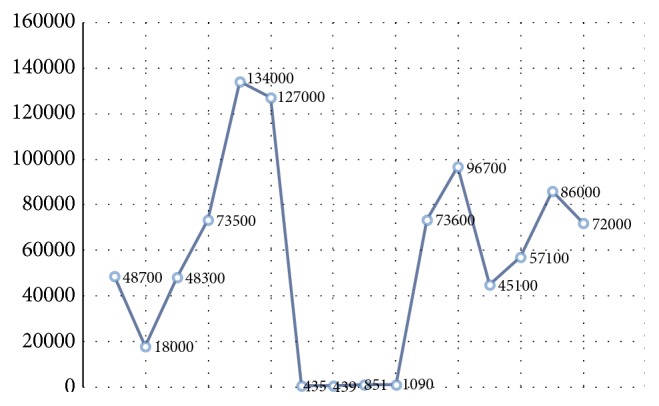
Comparison between IgE levels during application of omalizumab. The y-axis: serum IgE expressed in IU/ml; the x-axis: the dates on which these levels were taken. The decrease in IgE levels was clearly observed during the application of omalizumab; these increased after treatment was discontinued.

On April 2008, a 37-year-old male from Mexico City, with diagnosis of atopic dermatitis since he was 5 years old, presented to dermatology service and was treated with efalizumab 1 mg/kg/SC/weekly for two months. The patient received twelve doses of efalizumab, but the drug was suspended due to herpes zoster dermatitis (Figure 1), and treatment with acyclovir was indicated with adequate clinical response. Two months later, the patient developed generalized erythroderma, finding high levels of total serum IgE (48,700 UI/ml) and interleukins levels: TNF 39.7 pg/ml, IL-1*β* 5 pg/ml, IL-2R 1086 IU/ml, IL-6 11.9 pg/ml, and IL-8 8.66 pg/ml, showing increased levels of proinflammatory cytokines. He was sent to the service of clinical immunology and allergy, where he was diagnosed with HIES. To evaluate the pathway of IL-21, PBMCs of the patient were stimulated with rhIL-21 (militenyi) for 15 minutes and phosphorylation of STAT3 was evaluated by flow cytometry. The mAbs used were anti CD19-APC, anti-pSTAT3 (Y705)-PE, and isotype controls (mouse IgG1) from BD Pharmingen and Santa Cruz Biotechnology. Flow cytometry was performed on CYAN-ADP cytometer (Beckman Coulter). Cells were analyzed using Flow Jo 8.8 software (Tree Star). On September 2008, the clinical manifestations were dry skin and lichenified upper limbs and chest, generalized itching, peeling, and scratching scars, treated with hydroxyzine 10 mg every 8 hours, efalizumab, emollients, and general measures. On January 2009, the treatment was modified with monoclonal antibody omalizumab 300 mg every two weeks, with initial levels of total IgE 127,000 IU/ml. We made a new clinical assessment at 6 weeks; the patient presented clinical improvement with eczematous lesions on the chest and upper limbs and secondary hypochromic lesions and traces of scratching. Reassessing their total serum IgE levels in 439 UI/ml, we decided to increase omalizumab dose to 350 mg every two weeks. On May 2013, the patient presented skin lesions exacerbation with erythema, itching, eczema, and erythroderma. To 2013 the patient received 74 doses of omalizumab with stability of clinical manifestations. Eczema and total IgE levels were decreased even though with variations. The variations of CRP (C-reactive protein) do not correlate with the clinical manifestations of the patient and IgE levels (Figures 2 and 3). The patient decided to stop the treatment by personal decision.

## 3. Discussion

We present the case of a patient with HIES and skin manifestations treated with humanized recombinant monoclonal antibody, omalizumab. HIES is a rare primary immunodeficiency, characterized by high levels of serum IgE, recurrent skin abscesses, eczema, and pneumonia. Inherited patterns with AD, AR have been reported, but most HIES cases are sporadic. Mutations in the signal transducer and activator of transcription 3 gene (*STAT3*) are a major cause of AD.

**Figure 4:**
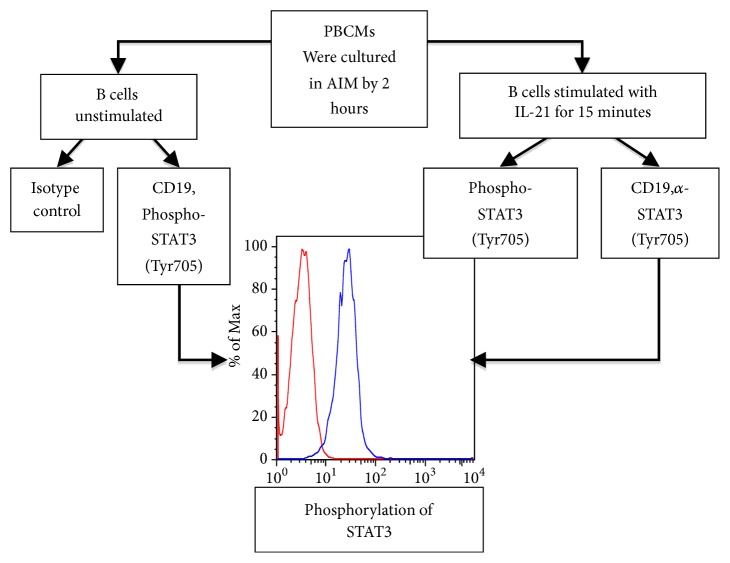
Evaluation of STAT3 phosphorylation by flow cytometry.

**Figure 5:**
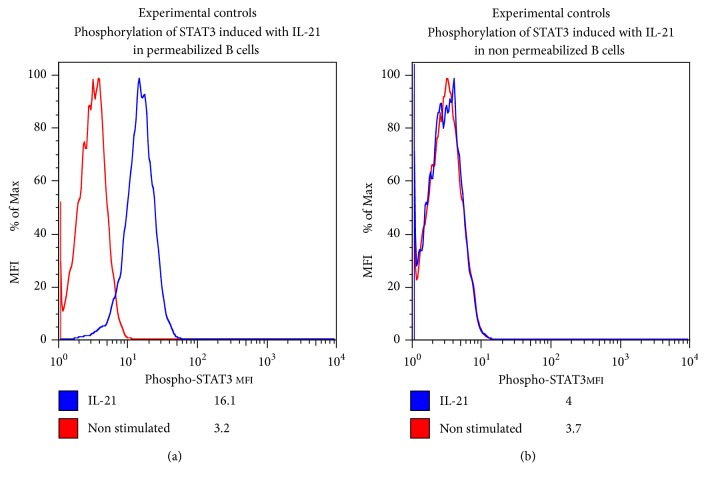
The patient showed low phosphorylation of STAT3 after 15 minutes of stimulation with rhIL-21; this image shows the comparison with experimental controls.

Interleukin 21 (IL-21) is needed for the differentiation of subsets of T cells CD4 the Th17 cells [5]. The patient showed low phosphorylation of STAT3 after 15 minutes of stimulation with rhIL-21. Experimental controls are appended (Figures 4 and 5). The low phosphorylation of STAT3 observed in HIES patients suggested that the activation and regulation of STAT3 might have an important role in T cell activation [6]. STAT3 regulates the subset Th_17_ cells which are important in inflammatory response to bacterial and fungal pathogens [7]. Defective Th17 responses are a common attribute of HIES cases with different genetic forms and sporadic cases [8]. Our patient had severe eczema and elevated levels of IgE that have never been reported in the world literature.

In the present case, dermatological findings led us to suspect HIES because of IgE levels and childhood onset-atopic dermatitis. Therefore, further investigations for HIES were done and the diagnosis was made by Grimbacher Criteria [9]. Omalizumab, the humanized recombinant monoclonal antibody against IgE, is known to result in marked reduction in serum free IgE and downregulation of IgE receptors on circulating basophiles. Omalizumab is used for the treatment of moderate to severe persistent asthma in adults and adolescents older than 12 years of age who have elevated levels of IgE and positive skin test to a perennial allergen. Several studies reported clinical improvement in patients with severe atopic eczema with high serum IgE level [10–13]. However, prospective studies and long-term follow-up are required to confirm the efficacy of omalizumab in HIES. In the present case, the clinical response was partial because the patient did not fulfill his treatment schedule. The reactivation of skin manifestations was related to the serum IgE increase.

## Abreviations

HIES: Hyper-IgE syndrome
IgE: Immunoglobulin E
AD: Autosomal dominant inheritance
AR: Autosomal recessive inheritance
HSV: Herpes simplex virus
DIDS: DOCK8 immunodeficiency syndrome
HPV: Human papillomavirus
MCV: Molluscum contagious virus
VZV: Varicella-zoster virus.
