# Erratum to “Partial and Transient Clinical Response to Omalizumab in IL-21-Induced Low STAT3-Phosphorylation on Hyper-IgE Syndrome”

**DOI:** 10.1155/2021/2361360

**Published:** 2021-01-31

**Authors:** Cesar Daniel Alonso-Bello, María del Carmen Jiménez-Martínez, María Eugenia Vargas-Camaño, Sagrario Hierro-Orozco, Mario Alberto Ynga-Durand, Laura Berrón-Ruiz, Julio César Alcántara-Montiel, Leopoldo Santos-Argumedo, Diana Andrea Herrera-Sánchez, Fernando Lozano-Patiño, María Isabel Castrejón-Vázquez

**Affiliations:** ^1^Immunology and Allergy Department, Centro Médico Nacional 20 de Noviembre, Instituto de Seguridad y Servicios Sociales de los Trabajadores del Estado, Mexico City, Mexico; ^2^Faculty of Medicine, Universidad Nacional Autónoma de México, Mexico City, Mexico; ^3^Dermatology Department, Centro Médico Nacional 20 de Noviembre, Instituto de Seguridad y Servicios Sociales de los Trabajadores del Estado, Mexico; ^4^Laboratorio de Inmunidad de Mucosas, Sección de Investigación y Posgrado, Escuela Superior de Medicina, Instituto Politécnico Nacional, Mexico City, Mexico; ^5^Immunodeficiencies Research Unit, Instituto Nacional de Pediatria, Secretaría de Salud, Mexico City, Mexico; ^6^Department of Molecular Biomedicine, CINVESTAV-IPN, Mexico City, Mexico

In the article titled “Partial and Transient Clinical Response to Omalizumab in IL-21-Induced Low STAT3-Phosphorylation on Hyper-IgE Syndrome” [1], authors Mario Alberto Ynga-Durand and Julio César Alcántara-Montiel were affiliated to “Universidad Nacional Autónoma de Mexico, Facultad de Estudios Superiores Zaragoza, Facultad de Medicina, Mexico,” which is incorrect.

The correct affiliation of Mario Alberto Ynga-Durand is “Laboratorio de Inmunidad de Mucosas, Sección de Investigación y Posgrado, Escuela Superior de Medicina, Instituto Politécnico Nacional, Mexico City, Mexico.” The correct affiliation for Julio César Alcántara-Montiel is “Department of Molecular Biomedicine, CINVESTAV-IPN, Mexico City, Mexico.”

The error was introduced during the production process of the article, and Hindawi apologises for causing this error in the article.

The corrected list of affiliations is shown in the authors' information above.
